# A small heat shock protein is essential for thermotolerance and intracellular survival of *Leishmania donovani*

**DOI:** 10.1242/jcs.157297

**Published:** 2014-11-01

**Authors:** Antje Hombach, Gabi Ommen, Andrea MacDonald, Joachim Clos

**Affiliations:** Bernhard Nocht Institute for Tropical Medicine, 20259 Hamburg, Germany

**Keywords:** *Leishmania*, Small heat shock protein, Alpha-crystallin, Stress tolerance, Infectivity, HSP23

## Abstract

*Leishmania* parasites must survive and proliferate in two vastly different environments – the guts of poikilothermic sandflies and the antigen-presenting cells of homeothermic mammals. The change of temperature during the transmission from sandflies to mammals is both a key trigger for the progression of their life cycle and for elevated synthesis of heat shock proteins, which have been implicated in their survival at higher temperatures. Although the functions of the main heat shock protein families in the *Leishmania* life cycle have been studied, nothing is known about the roles played by small heat shock proteins. Here, we present the first evidence for the pivotal role played by the *Leishmania donovani* 23-kDa heat shock protein (which we called HSP23), which is expressed preferentially during the mammalian stage where it assumes a perinuclear localisation. Loss of HSP23 causes increased sensitivity to chemical stressors and renders *L. donovani* non-viable at 37°C. Consequently, HSP23-null mutants are non-infectious to primary macrophages *in vitro*. All phenotypic effects could be abrogated by the introduction of a functional HSP23 transgene into the null mutant, confirming the specificity of the mutant phenotype. Thus, HSP23 expression is a prerequisite for *L. donovani* survival at mammalian host temperatures and a crucial virulence factor.

## INTRODUCTION

The obligate intracellular protozoan parasites of the genus *Leishmania* are causative for the various forms of Leishmaniasis. The clinical manifestations range from self-healing skin lesions (cutaneous leishmaniasis) to lethal, generalised infections of the reticulo-endothelial system (visceral leishmaniasis). The infections are found on four continents with an estimated annual incidence of between 900,000 and 1.8 million (Alvar et al., 2012). *Leishmania* spp. undergo two life cycle stages – the extracellular promastigote stage in the insect vector, and the intracellular amastigote form, in the phagosomes of infected macrophages. Thus, the parasites colonise two very diverse environments in the course of their life cycle. The transmission of *Leishmania* spp. from a poikilothermic insect vector to a homeothermic mammalian host is linked to an increase in ambient temperature. The increase in temperature induces a heat stress response that is directly associated with the differentiation of the parasite (Krobitsch et al., 1998); the combination of an increased temperature and the acidic pH in the phagosomes of infected macrophages causes conversion from the promastigote to the amastigote stage (Bates et al., 1992), which is pivotal for parasite survival.

The heat shock response is a universal reaction that protects cells and organisms against elevated temperatures. This reaction correlates with an increased synthesis of so-called heat shock proteins (HSPs), whose synthesis is also induced by other stressors, such as metal ions, low pH and oxidative cell stress (Morimoto et al., 1990; Vonlaufen et al., 2008). The expression of HSPs represents a fast and efficient cellular response to various stresses.

HSPs are highly conserved proteins and present in all cell types and organisms. Based on their molecular masses and primary structures, HSPs are divided into different families – HSP110, HSP100, HSP90, HSP70, HSP60, HSP40, HSP10 and the small HSPs (Feder and Hofmann, 1999; Smith et al., 1998). Under cell stress conditions HSPs interact with denatured proteins and inhibit the formation of cytotoxic protein aggregates, thereby maintaining the protein homeostasis of a cell (Bukau et al., 2006; Feder and Hofmann, 1999; Mayer and Bukau, 1999). Under physiological conditions, most HSPs possess chaperone activity. They interact with many highly diverse client proteins to support and control maturation, activation, translocation or degradation of those client proteins.

The genomes of *Leishmania* spp. encode the full set of HSP families (Ommen and Clos, 2009), and these are either synthesised constitutively during both life cycle stages (Rosenzweig et al., 2008) or their abundance increases during amastigote differentiation, correlating with the stress response of the parasite (Hübel et al., 1995; Krobitsch, 1998; Schlüter et al., 2000; Zamora-Veyl et al., 2005). The best characterised HSP in *Leishmania* so far is HSP100. Chaperones of the HSP100 family are known to confer inducible thermotolerance in organisms like bacteria, yeast and higher plants (Lindquist, 1992; Parsell et al., 1994; Parsell and Lindquist, 1994; Sanchez et al., 1992; Schirmer et al., 1996). The phenotypic analyses of *L. donovani* and *L. major* HSP100-null mutants have indicated that the *Leishmania* homologues are not involved in thermotolerance. Nevertheless, the HSP100-null mutants proved unable to proliferate and survive inside mouse macrophages after *in vitro* infection and, in the case of the *L. major* HSP100-null mutant, to establish an infection in BALB/c mice (Hübel et al., 1997; Krobitsch et al., 1998; Krobitsch and Clos, 1999). This is probably due to *Leishmania* HSP100 being crucial for the proper sorting of proteins into exosomes, and thus for the immune modulatory function of these export vesicles (Silverman et al., 2010).

Another HSP whose function is crucial for the maintenance of both parasitic life cycle stages is HSP90 (Hombach et al., 2013; Wiesgigl and Clos, 2001). This chaperone is an essential, conserved and constitutively expressed HSP that is associated with the maturation and activation of a variety of signalling client proteins, such as kinases, transcription factors and hormone receptors (Buchner, 1999; Nathan and Lindquist, 1995; Ochel et al., 2001; Picard, 2002; Pratt and Toft, 2003). HSP90 is the central member of the so-called ‘foldosome’ complex. Further members of this complex are other chaperones, such as HSP70 and HSP40, and co-chaperones such as Sti-1 (HOP) and P23 (Sba1). The correct interplay of the foldosome members facilitates the maturation and activation of essential client proteins (Johnson, 2012; Johnson and Brown, 2009; Prodromou, 2012).

The HSP70 and HSP40 families are numerous and diverse in *Leishmania* spp. However, despite this, very little is known about the control and roles of these genes (Hombach and Clos, 2014).

Small HSPs (sHSPs) represent the most widespread, extremely heterogeneous family of molecular chaperones. Nevertheless, the sHSPs share some characteristic features that include: (1) a conserved α-crystallin domain (ACD) of ∼90 residues, (2) a small molecular mass ranging from 12–43 kDa, (3) the ability to form large oligomers, (4) a dynamic quaternary structure, (5) induction at stress conditions, and (6) chaperone activity (Haslbeck et al., 2005; Horwitz, 2003). The ACD is named after the human lenticular protein α-crystallin, which prevents protein aggregation in the human eye. ACDs are flanked by variable N- and C-terminal domains in sHSP family members. The sHSPs have a multitude of crucial functions under normal and pathological conditions. They represent molecular chaperones with strong anti-aggregation properties, interact with the major cytoskeletal components to regulate their assembly and protect cells against various stresses by their ability to prevent damage induced by these stressors and/or their ability to interfere with key pro-apoptotic proteins (Acunzo et al., 2012; Garrido et al., 2012; Wettstein et al., 2012).

It is well known that there is a connection between the large HSPs of various parasitic organisms and their virulence potential (Banumathy et al., 2003; Hombach et al., 2013; Hübel et al., 1997; Pallavi et al., 2010). However, the functions of sHSPs and their potential roles in parasite pathogenicity are so far mostly unknown. One study of HSP21 from the pathogenic fungus *Candida albicans* indicates that sHSPs are directly involved in the adaption to specific stresses and are crucial for the pathogenicity of this fungus (Mayer et al., 2012). Five potential sHSPs have already been identified in the intracellular parasite *Toxoplasma gondii*. Those are located in different cellular compartments and are differentially expressed during the life cycle of the parasite. All of them exhibit the characteristic properties of sHSPs, such as oligomeric structure and chaperone activity (de Miguel et al., 2009). For *Plasmodium*, it has been suggested that a sHSP, HSP20, is involved in sporozoite cellular adhesion and migration (Montagna et al., 2012).

So far nothing is known about the functions of sHSPs in *Leishmania* spp. For *L. mexicana* panamensis, it has been shown that after heat stress the synthesis of small proteins (22–27 kDa) is increased (Hunter et al., 1984). However, none of these proteins had previously been identified. In this study, we report the identification of three ACD-containing proteins and the characterisation of an atypical 23-kDa sHSP in *L. donovani*. Owing to its molecular mass, we named the corresponding gene *HSP23* (LinJ.34.0230). By molecular analysis, we demonstrated that the expression of this newly identified sHSP is induced during stage conversion, is involved in the *L. donovani* adaption to specific stresses and is essential for the infectivity in a murine macrophage model. This work represents the first detailed description of a sHSP in kinetoplastid protozoa.

## RESULTS

### Kinetoplastida possess atypical sHSPs

**Fig. 1.**
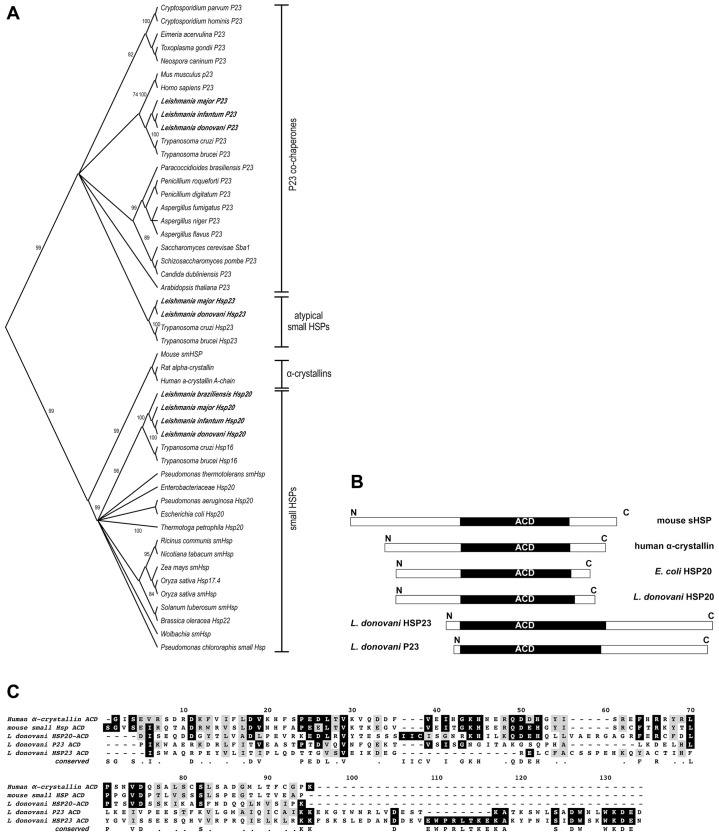
**Phylogenetic analysis of *Leishmania* α-crystallin proteins.** (A) Sequence alignment and tree building were done using the neighbour-joining algorithm and bootstrap analysis (1000 repeats). *Leishmania* proteins are highlighted by bold print. The clades of P23-like proteins, α-crystallins, sHSPs and atypical sHSPs are marked on the right. (B) Schematic alignment of three *L. donovani* α-crystallin proteins with typical representatives of this protein family. Note the relative positions of the α-crystallin domain (ACD). (C) Amino acid sequence alignment of the ACDs from human α-crystallin, mouse small HSP, and 3 *L. donovani* ACD proteins. Sequence identities are shown on a black background; similar amino acids shown on a grey background; conserved residues are shown beneath the sequences.

Because virtually nothing was known about the roles and functions of α-crystallin-domain-containing proteins (ACDPs) in kinetoplastid protozoa, we first performed a search of the Tritryp database (http://tritrypdb.org/tritrypdb/), using the human P23 co-chaperone sequence (also known as prostaglandin E synthase 3) and the BLAST algorithm. We identified three distinct putative coding sequences with ACD signature, namely LinJ.29.2560 (which we termed HSP20), LinJ.35.4540 (which we termed P23) and LinJ.34.0230 (which we termed HSP23), in accordance with their position within a phylogenetic tree of ACDPs (Fig. 1A). *Leishmania* HSP20 proteins grouped with bacterial and plant small HSPs, whereas the putative P23 co-chaperone grouped with mammalian and fungal P23 co-chaperones.

The kinetoplastid HSP23 proteins appear to originate from P23 proteins, but represented a separate lineage (Fig. 1A). They and the putative kinetoplastidic P23 orthologs also showed an atypical domain arrangement, in that their ACDs were located very close to the N-termini (Fig. 1B), which is different to the canonical structure of small HSPs and P23 proteins, which carry long N-terminal domains in front of their ACDs. We also compared the sequences of the ACDs only and found them considerably diverged, underscoring the generally low degree of sequence conservation in ACDPs (Fig. 1C). We therefore decided to investigate both *L. donovani* P23 (A.H. and G.O., unpublished data) and the atypical HSP23 (this paper).

### HSP23 is a stress-inducible protein in *Leishmania*

To detect HSP23 in *Leishmania*, we first raised specific antibodies against recombinantly expressed protein. The HSP23 coding sequence was inserted into pJC45 expression vector (Schlüter et al., 2000) and expressed as His_10_ fusion protein in *E. coli*. Total extracts (supplementary material Fig. S1A) were purified using a Ni^2+^ metal affinity resin (Clos and Brandau, 1994), resulting in a pure His_10_–HSP23 fusion protein (supplementary material Fig. S1A). The purified antigen was then mixed with Titermax Gold adjuvant and used to immunise laying hens. Antibodies (IgY) were purified from egg yolk and tested in western blot analyses. The antibodies detected 0.1 ng of recombinant HSP23 at a 500-fold dilution (supplementary material Fig. S1B) and natural HSP23 in lysates from three *Leishmania* species (supplementary material Fig. S1B).

**Fig. 2.**
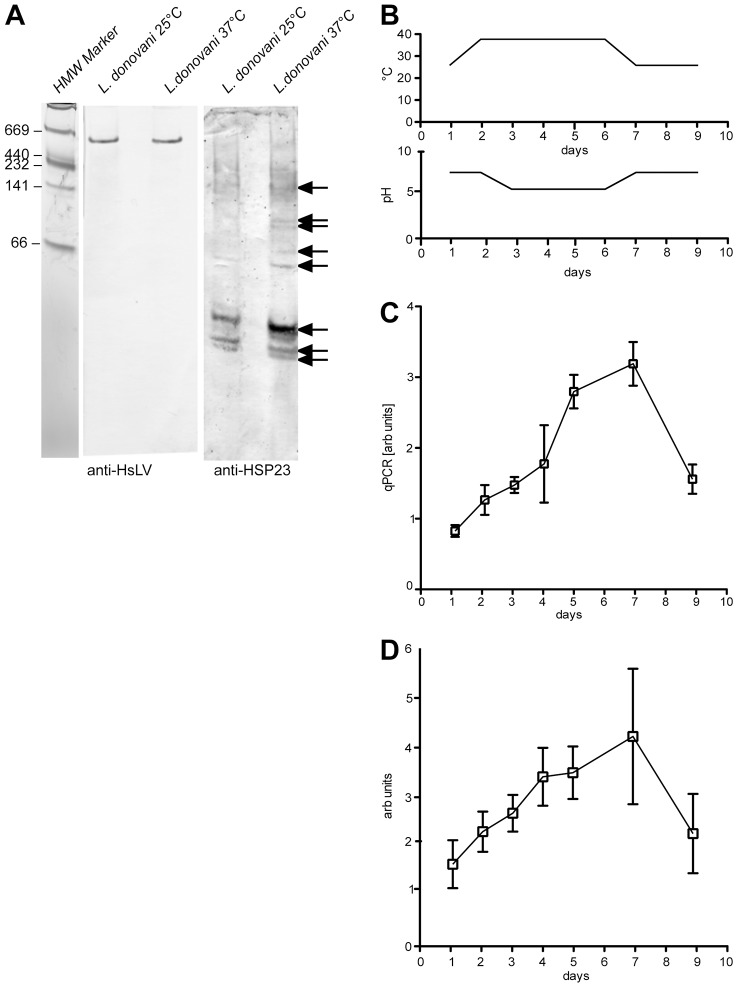
**Expression kinetics of HSP23.** Native PAGE analysis of *L. donovani* log phase promastigotes grown at 25°C (A, lane 1 and 3) and heat-shocked promastigotes incubated for 24 h at 37°C (A, lane 2 and 4) showed that the abundance of HSP23 oligomers (arrows), but not of the HslV oligomer, increases after heat-stress. To determine the expression profile of HSP23 during the life cycle of *L. donovani*, a temperature and pH-dependent axenic stage differentiation was performed. Temperature and pH profiles are shown in B. The amounts of HSP23 RNA (arbitrary units) were measured by RT-qPCR and plotted against time (C). HSP23 protein abundance (arbitrary units) was quantified by western blotting and image analysis and plotted against time (D).

Small HSPs are known to associate into oligomeric complexes. Therefore, we investigated the native size of *Leishmania* HSP23 using native gradient PAGE (Andersson et al., 1972; Ommen et al., 2010). The results showed that the majority of HSP23 was in a presumably monomeric form. However, several larger species can be seen, ranging from ∼60 kDa to ∼160 kDa, likely representing HSP23 oligomeric complexes that had increased abundance at higher cultivation temperatures (Fig. 2A, lanes 3 and 4). The formation of such complexes is characteristic of small HSPs. As loading control, we used the HslV peptidase of *L. donovani*. HslV is constitutively expressed in *L. donovani* and exists primarily in a multimeric complex (Chrobak et al., 2012). Accordingly, the HslV signal is not increased in the samples from heat-shocked promastigotes, confirming the observed induction of HSP23 as specific (Fig. 2A, lanes 1 and 2).

Because we suspected that HSP23 played a role in the *Leishmania* stress response, we also studied the expression kinetics of *HSP23* during an *in vitro* life cycle (Fig. 2B). Promastigotes of *L. donovani* grown at 25°C and pH 7.0 (day 1) were exposed to 37°C (day 2) and shifted to a pH 5.5 (day 3) to induce *in vitro* amastigote differentiation (day 4 to 7). At day 7, the axenic amastigotes were seeded into pH 7.0 medium and shifted back to 25°C to facilitate amastigote-to-promastigote conversion (day 9). Samples were taken at relevant time points and tested for HSP23 RNA levels (Fig. 2C) and HSP23 protein abundance (Fig. 2D; supplementary material Fig. S1C). Both RNA levels and protein abundance increased synchronously during the axenic amastigote stage but returned to normal upon reaching the promastigote stage again. We conclude that HSP23 is a stress-inducible protein with a three-fold higher abundance in early amastigotes.

### HSP23 changes abundance and subcellular localisation during its life cycle

**Fig. 3.**
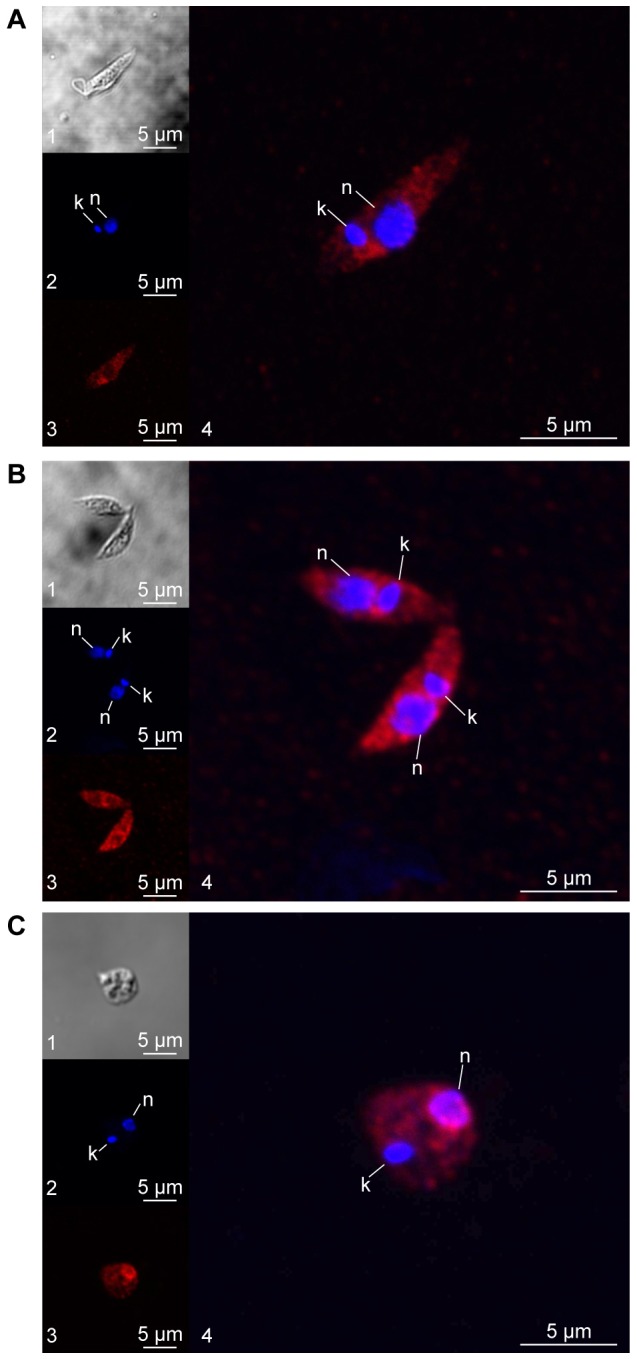
**Subcellular localisation of HSP23 in *L. donovani* life cycle stages.** Log phase promastigotes at 25°C (A) or 37°C (B), and axenic amastigotes (C), were stained with chicken anti-HSP23 antibody (1∶100) and DAPI (1∶25). Images were taken by confocal laser microscopy and in differential interference contrast (DIC). Representative cells from each culture were visualised with DIC (panels A1, B1, C1), DAPI (panels A2, B2, C2), anti-HSP23 antibody (panels A3, B3, C3) and DAPI and anti-HSP23 antibody overlays (panels A4, B4, C4). k, kinetoplast; n, nucleus.

Using the HSP23-specific antibodies in confocal laser microscopy, we compared its localisation under three different cultivation conditions, (1) promastigotes at 25°C (Fig. 3A), (2) heat-stressed (37°C) promastigotes (Fig. 3B), and (3) axenic amastigotes (Fig. 3C). At 25°C, HSP23 had a cytoplasmic localisation (Fig. 3A, panels 3 and 4). At 37°C, the staining intensity increased, in keeping with the heat-inducibility we observed in Fig. 2D. In addition, we found a minor shift to the vicinity of the nucleus (Fig. 3B, panels 3 and 4). In axenic amastigotes (Fig. 3C, panels 3 and 4), HSP23 was found almost exclusively in a perinuclear localisation, indicting a role in nuclear processes at mammalian temperatures. These results further indicate a participation of HSP23 in the cellular stress response.

### HSP23 and HSP70 colocalise under heat stress

**Fig. 4.**
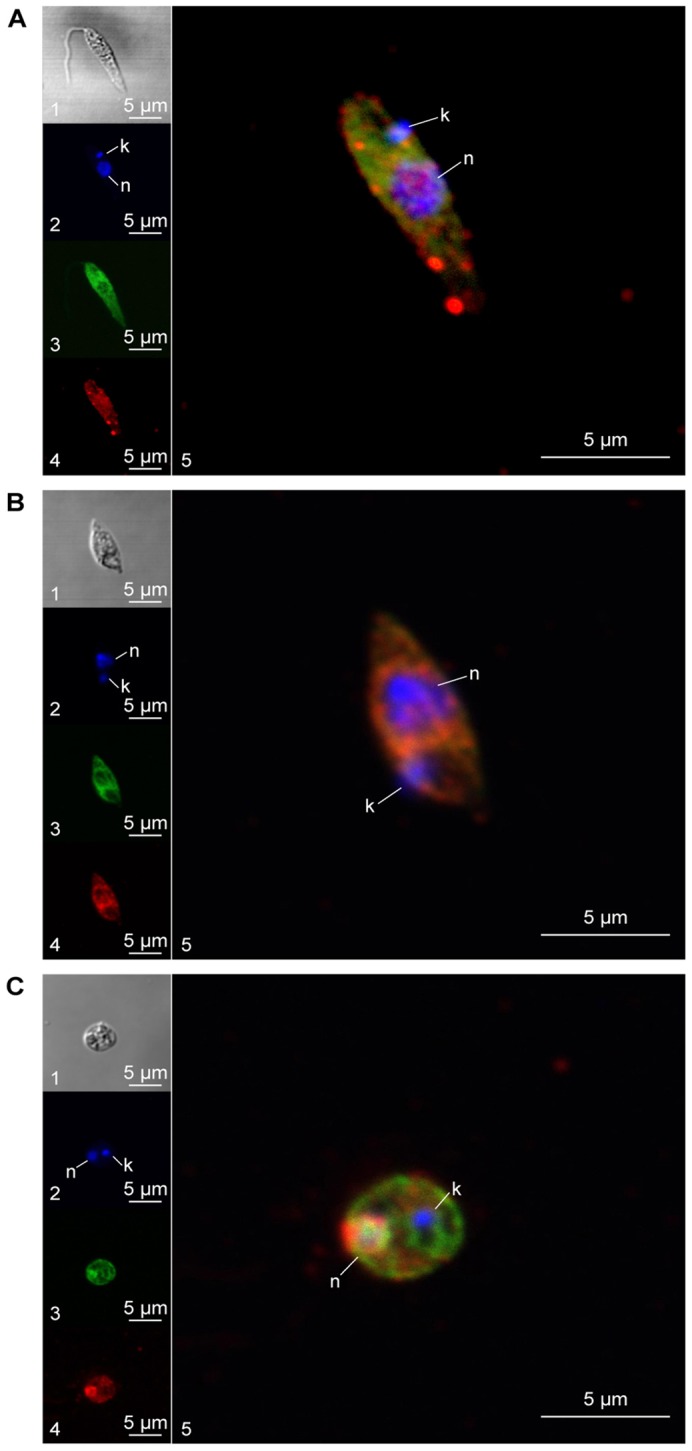
**Comparative subcellular localisation analysis.** Log phase promastigotes at 25°C (A) or 37°C (B), and axenic amastigotes (C) were stained with mouse anti-HSP70 antibody (1∶125), chicken anti-HSP23 antibody (1∶100) and DAPI (1∶25). Images were taken by confocal laser microscopy and in differential interference contrast (DIC). Representative cells from each culture were visualised with DIC (panels A1, B1, C1), DAPI (panels A2, B2, C2), anti-HSP70 antibody (panels A3, B3, C3), anti-HSP23 antibody (panels A4, B4, C4) and DAPI, HSP70 and HSP23 overlays (panels A5, B5, C5). k, kinetoplast; n, nucleus.

To investigate possible interactions of HSP23 with the major chaperones of *Leishmania*, we also performed colocalisation studies for HSP23 with HSP70, HSP90 and HSP100, respectively, using the same culture forms as above. Neither HSP90 nor HSP100 showed discernible colocalisation with HSP23 (supplementary material Fig. S2), suggesting that neither chaperone interacts selectively with this small HSP. The colocalisation studies with HSP70, however, revealed a convergence of both proteins around the nucleus under heat stress (Fig. 4B, panel 5). Promastigotes kept at 25°C showed no such colocalisation patterns (Fig. 4A, panel 5). Partial colocalisation in a perinuclear position was also observed for the axenic amastigotes (Fig. 4C, panel 5). Taken together, the results suggest a convergence of HSP23 with cytoplasmic HSP70 at mammalian tissue temperatures.

### Generation of HSP23-null mutants in *L. donovani*

**Fig. 5.**
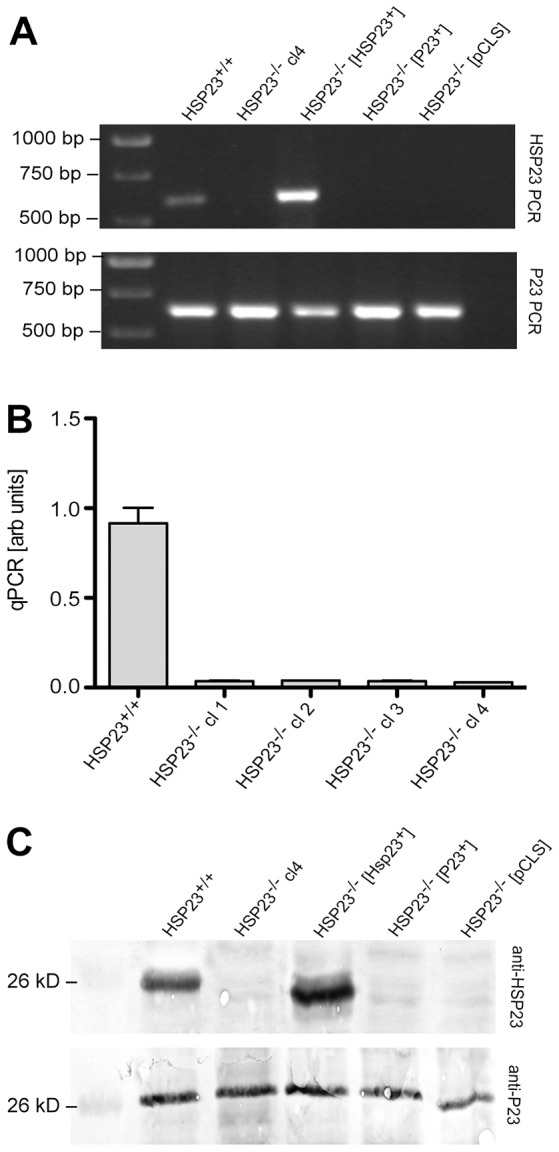
**Verification of *L. donovani* HSP23-null mutants.** The genomic (g)DNAs from *L. donovani* strains HSP23^+/+^ (wild type), HSP23^−/−^, HSP23^−/−^HSP23^+^, HSP23^−/−^P23^+^ and HSP23^−/−^ pCLS were used as templates for a P23-gene-specific PCR (A, top panel) to test the quality of the gDNAs. The same gDNAs were then used as template for a HSP23-specific PCR (A, bottom panel). The position of size markers (bp) are shown on the left. HSP23 mRNA levels (arbitrary units) were quantified by RT-qPCR for HSP23^+/+^ and for the four suspected null mutant clones, HSP23^−/−^ cl1–cl4 (B). Lysates from *L. donovani* strains HSP23^+/+^, HSP23^−/−^, HSP23^−/−^HSP23^+^, HSP23^−/−^ P23^+^, HSP23^−/−^ pCLS were separated by SDS-PAGE, subjected to western blotting and probed with anti-P23 antibody (1∶200) (C, top panel) or anti-HSP23 (1∶500) (C, bottom panel).

Given that *HSP23* is expressed predominantly at higher temperatures, we expected that HSP23-null mutants might be viable at low cultivation temperatures. Therefore, we targeted the *HSP23* alleles with specific replacement constructs (supplementary material Fig. S3A), using a sequential homologous recombination strategy (Krobitsch and Clos, 2000) (supplementary material Fig. S3B). Using the null mutants as background, we introduced HSP23 and P23 transgenes as episomes (supplementary material Fig. S3C). The genotypes were then verified in three ways: (1) by HSP23-specific PCR from genomic DNA (Fig. 5A), (2) by quantitative real-time RT-PCR (RT-qPCR) of four individual null mutant clones (Fig. 5B), and (3) by western blot analysis using HSP23-specific IgY (Fig. 5C).

The ∼600-bp HSP23 coding sequence could be amplified both from wild-type and from HSP23^−/−^ [HSP23^+^] DNA (Fig. 5A, upper panel, lanes 1, 3). The HSP23^−/−^ gDNA (lane 2) and the control strains carrying a P23 transgene (lane 4) or the empty pCLS vector (lane 5) did not carry HSP23 coding sequences. We also amplified P23 coding sequences from the same gDNA samples to verify their quality. All gDNA gave rise to P23 amplicons (Fig. 5A, lower panel). The lack of HSP23 coding sequences was also evident from the results of a semi-quantitative, HSP23-specific real-time PCR using cDNAs from wild-type and null mutant clones (Fig. 5B). All four putative null mutant clones were devoid of HSP23-specific RNA, corroborating the qualitative PCR in Fig. 5A. Finally, we verified the wild-type, null mutant and null-mutant-derived transgenic strains by western blotting using HSP23-specific IgY. Wild-type and null mutants with the HSP23 transgene showed HSP23 protein expression (Fig. 5C, upper panel, lanes 1, 3). Neither the null mutant nor its derivatives carrying the P23 transgene or the empty pCLS vector showed any signal for HSP23 protein (lanes 2, 4, 5). The quality of the protein samples was verified by western blotting using P23-specific IgY (Fig. 5C, lower panel). Thus, using three independent methods, we can verify the HSP23-null mutants and their derivatives.

### HSP23 is essential for *Leishmania* stress tolerance

**Fig. 6.**
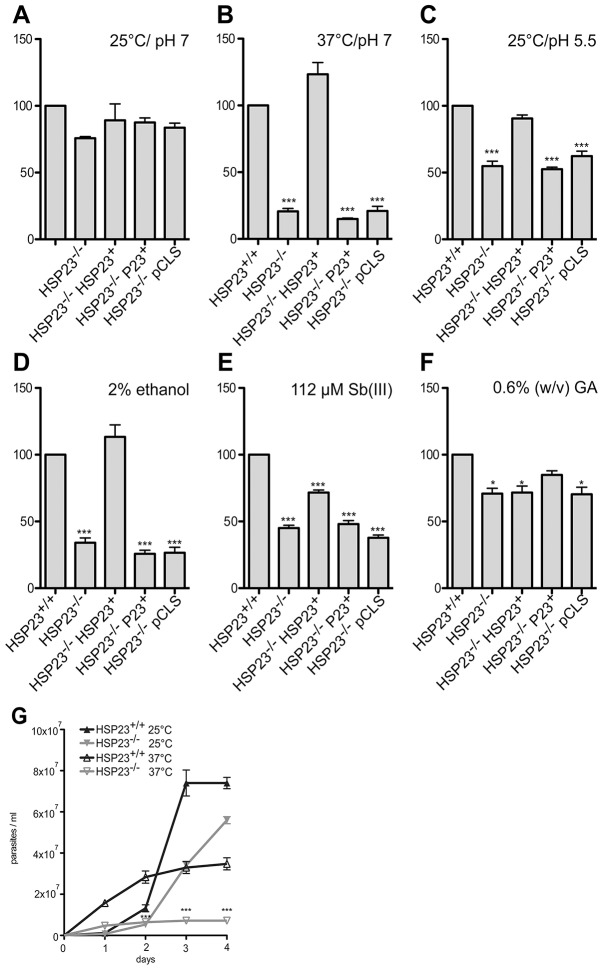
**Proliferation of HSP23-null mutants under stress.** 1×10^5^ cells ml^−1^ were seeded into 10 ml of supplemented M199+ medium and grown for 4 days. Cell density on day 4 was then calculated as a percentage of wild-type (HSP23^+/+^) cell density (set at 100%). Parasites were grown at 25°C and pH 7.0 (A), 37°C and pH 7.0 (B), 25°C and pH 5.5 (C). Additional cultures were grown at 25°C and pH 7.0 with the addition of 2% ethanol (D), 112 µM antimonyl tartrate [Sb(III)] (E) or 0.6% (w/v) geldanamycin (GA). The cell density of wild-type cells (HSP23^+/+^) and HSP23-null mutants (HSP23^−/−^) at 25°C and pH 7.0 or 37°C and pH 7.0 was analysed for 4 days (G). The *y*-axis shows percentage of wild-type growth (A–F) or cell density (×10^7^) (G). *P<0.05; ***P<0.001 (Mann–Whitney U-test).

In order to investigate the phenotype of the null mutant and its derivatives compared with wild-type *L. donovani*, we performed growth experiments with and without cellular stress. At ideal *in vitro* growth conditions (25°C, pH 7.0), the null mutant showed no significant growth defect (Fig. 6A,G), in keeping with its limited expression in promastigotes.

At 37°C, corresponding to the mammalian tissue temperature, the HSP23-null mutant was not viable and did not proliferate (Fig. 6B,G). This temperature-sensitive phenotype was complemented by the HSP23 transgene, but not by P23 overexpression or by the empty expression plasmid pCLS (Fig. 6B). The temperature sensitivity was highly significant and was corroborated by scanning electron microscopy, which showed that the HSP23^−/−^ parasites were badly damaged at this temperature (data not shown). The second stimulus for promastigote-to-amastigote conversion, an acidic milieu, also affects the HSP23-null mutant stronger than wild-type or HSP23-reconstituted null mutants (Fig. 6C). Again, only HSP23 transgenes can abrogate this pH-sensitive phenotype.

We also tested the ethanol tolerance of the null mutant because this stimulus causes an unfolded protein stress and can replace heat shock in the *in vitro* amastigote differentiation (Barak et al., 2005). At the IC_50_ (2% v/v), ethanol caused a growth arrest for the HSP23-null mutant and for the P23 and vector control strains. Only wild-type and the HSP23-reconstituted null mutants showed a sustained 50% growth compared with untreated cells (Fig. 6D). Although Barak et al. (Barak et al., 2005) have observed a morphological change at 5% ethanol, the wild-type cells did not change their cell length at 2% ethanol. However, the HSP23-null mutant reacted to this ethanol concentration with a shortening of the cell body and a reduction in the flagellum length (supplementary material Fig. S4B), however, without any visible cell damage.

HSP23 also protects against another stressor, trivalent antimony (Sb^3+^), the active principle of pentavalent antimony anti-leishmanials. The HSP23-null mutant showed a markedly reduced growth compared to the wild-type at 112 µM Sb^3+^, the IC_50_ for this compound (Fig. 6E). HSP23 expression in the null mutant partially abrogated this defect. Here too, exposure of the null mutants caused a shortening of the cell body and the flagellum (supplementary material Fig. S4C). The HSP90 inhibitor geldanamycin affects the null mutant marginally stronger than the wild-type; however, no restorative effect was seen with HSP23 add-back strain (Fig. 6F). Interestingly, overexpression of P23, which is implied in resistance to geldanamycin (Forafonov et al., 2008) restored growth of the HSP23-null mutant exposed to geldanamycin.

These data imply that HSP23 protects *L. donovani* against cell stress, namely elevated temperature, unfolded protein stress and redox stress. Loss of HSP23 sensitises *L. donovani* to all these stresses and, in particular, results in a temperature-sensitive phenotype. The structurally related P23 cannot alleviate this phenotype and is clearly functionally different from HSP23.

### HSP23-null mutants fail to infect macrophages

**Fig. 7.**
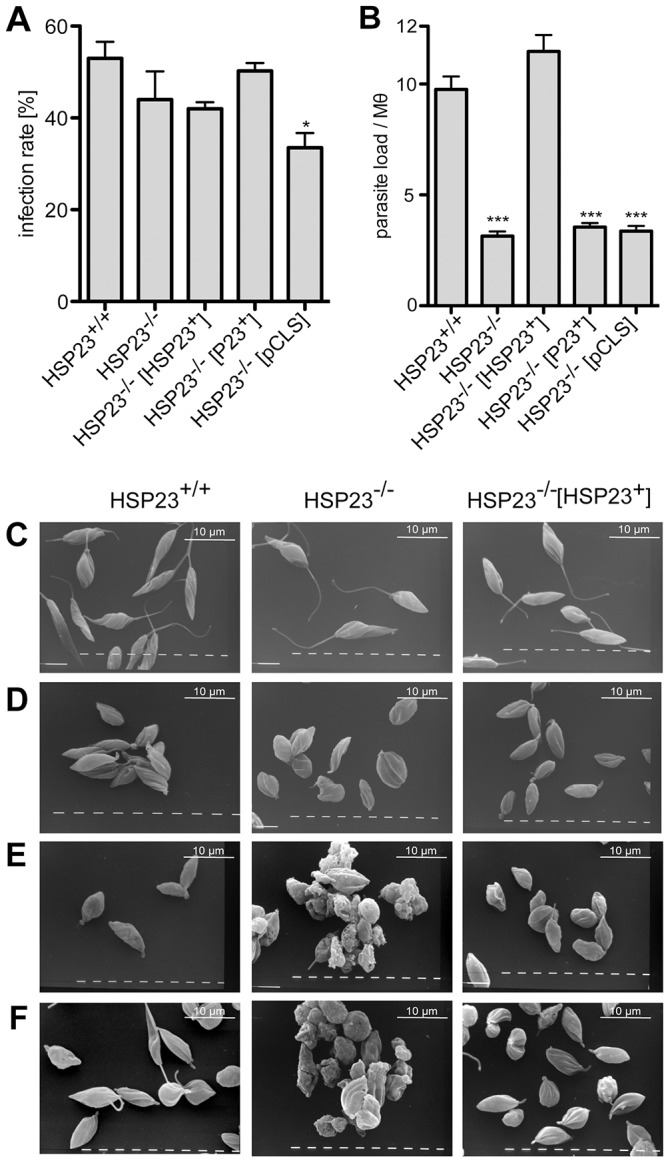
**Infectivity of HSP23-null mutants *in vitro*.** Bone-marrow-derived macrophages (Mθ) were infected with HSP23^+/+^, HSP23^−/−^, HSP23^−/−^HSP23^+^, HSP^−/−^P23^+^ and HSP23^−/−^ pCLS parasites at a 1∶10 ratio. After 4 h, free parasites were removed and the infected macrophage cultures were further incubated at 37°C under 5% CO_2_ for 44 h. Cells were fixed and stained with DAPI. 400 macrophages per infection experiment were checked by fluorescence microscopy for calculation of the infection rate (A, percentage of infected Mθ) and parasite load (B, number of parasites per Mθ). (C–F) Scanning electron microscopy images of different stages during *in vitro* axenic amastigote differentiation and promastigote re-differentiation. *L. donovani* HSP23^+/+^, HSP23^−/−^ and HSP23^−/−^ HSP23^+^ log phase promastigotes (C), heat-shocked promastigotes (D), axenic amastigotes (E) and re-converted promastigotes (F). *P<0.05; ***P<0.001 (Mann–Whitney U-test).

The same set of five *L. donovani* strains were also tested for their ability to infect bone-marrow-derived macrophages *in vitro*. Parasites were added to primary macrophages at a 10∶1 ratio. After 4 h, free parasites were washed off, after which the incubation was continued for another 44 h. Cells were then fixed and stained with DAPI for fluorescence microscopy evaluation of infection rate (percentage of infected macrophages) and parasite load (parasites per infected macrophage). The loss of HSP23 affected overall infection rate only slightly (and insignificantly) (Fig. 7A), indicating equal rates of uptake by the macrophages. The loss of HSP23, however, impaired the ability of *L. donovani* for intracellular survival and/or proliferation (Fig. 7B). Although wild-type parasites averaged at ten amastigotes per host cell, the HSP23-null mutant gave only a marginal parasite load of three amastigotes per host cell. Adding a HSP23 transgene brought the parasite load back up to >10 amastigotes per macrophage, confirming that the loss of HSP23 is causative for the loss of infectivity. Neither the P23 transgene nor the vector alone had any positive effect on the parasite counts. This result is in perfect agreement with the observed loss of temperature tolerance in the null mutant.

We also tested the ability of wild-type, HSP23^−/−^ mutant and the HSP23^−/−^ [HSP23^+^] add-back strains to survive an *in vitro* stage differentiation regimen initiated by a 24-h heat shock at 37°C followed by incubation at 37°C and pH 5.5. Fig. 7C–E shows scanning electron micrographs of the strains under those conditions. All strains show normal morphology at 25°C (Fig. 7C). After 24 h at 37°C, the wild-type, add-back and HSP23-null mutant strains showed a shortening of the flagella and the cell bodies (Fig. 7D). After 4 days at 37°C and 3 days at acidic pH, the wild-type and add-back strains displayed the typical shape of axenic amastigotes, including clustering and small, ovoid cell bodies. By contrast, nothing remained of the HSP23-null mutant but cell debris (Fig. 7E). In addition, attempts to regrow promastigotes from the axenic amastigote cultures failed completely for the HSP23^−/−^ strain, whereas the wild-type and add-back strains underwent successful amastigote-to-promastigote conversion (Fig. 7F).

We conclude that HSP23 is essential for survival of *L. donovani* at the temperatures of humans and other mammalian hosts, and that the HSP23^−/−^ strain gives a true temperature-sensitive phenotype.

## DISCUSSION

Many organisms survive and thrive in highly challenging environments. Even more so, many organisms must cope with rapidly changing living conditions. Among the latter are plants, sessile marine organisms, and also parasitic organisms, such as *Leishmania* spp. Establishing and maintaining adequate stress tolerance is therefore a key for evolutionary success and the population of ecological niches.

Traditionally, stress tolerance is mostly seen in the context of stress proteins and their functions (Sanchez and Lindquist, 1990; Sanchez et al., 1992). The primary stress tolerance effectors have been identified by means of reverse genetics, genetic complementation studies and systems biology approaches, and belong to the HSP70, HSP100 and sHSP families of chaperones.

The HSP100 or ClpB chaperones are absent from vertebrates, but ubiquitous in eubacteriae, plants, fungi and protozoa – i.e. in non-motile organisms (Schirmer et al., 1996). HSP100 interacts with HSP70 and HSP40 to restore stress-sensitive protein complexes. Its expression has been found to correlate with inducible thermotolerance in yeast, fungi, bacteria and plants. It has also been suggested that it is the effector of thermotolerance in *Leishmania* (Hübel et al., 1995; Hübel et al., 1997). This role, however, is minor at best (Krobitsch and Clos, 1999) because the real impact of HSP100 lies in the proper sorting of exosomal payload proteins (Silverman et al., 2010).

Overexpression of HSP70 has also been seen to be correlated with or to result in increased stress resistance in insects, vertebrates and plants (Feder and Hofmann, 1999). At this point, we cannot exclude a similar role in *Leishmania* owing to experimental constraints. However, activation of HSP70 synthesis is only transient and does not result in significantly increased abundance (Brandau et al., 1995). However, HSP70, like other heat shock proteins, is subject to stress-inducible phosphorylation, which might impact on its activity during different life cycle stages (Morales et al., 2010).

Our data clearly show that at least one sHSP, HSP23, plays a decisive role in the adaption to the temperatures found in mammalian tissue. Loss of HSP23 renders *L. donovani* incapable of surviving at 37°C and consequently non-infectious to *ex vivo* macrophages kept at this temperature. The data showed that high temperatures not only abrogate growth of HSP23-null mutants but outright destroy the cells, meaning that the HSP23^−/−^ strain gives a bona fide temperature-sensitive phenotype. Consequently, *in vitro*, axenic amastigote conversion is also abrogated for the HSP23^−/−^ parasites. Exposing the null mutants to the differentiation conditions results in cell destruction.

This temperature-sensitive phenotype of *L. donovani HSP23^−/−^* is highly reminiscent of *Candida albicans* HSP21-null mutants. Lack of this sHSP renders the pathogenic fungus non-infectious and incapable of proliferation at mammalian tissue temperatures, likely by interfering with stress-inducible signal transduction pathways (Mayer et al., 2012). By contrast, deletion of the sHSP BAG1/HSP30 in *Toxoplasma gondii* does not result in any phenotypical changes in either stage of that parasite (Bohne et al., 1998). A different but essential role is played by the *Plasmodium berghei* HSP20. Loss of the protein results in impaired migration inside the host tissue by the infecting sporozoite stage, likely impeding the liver stage formation, which is a prerequisite for a successful infection (Montagna et al., 2012).

Temperature tolerance is not the only trait affected by the loss of *HSP23*. Sensitivity to chemical stressors, such as ethanol and semi-metal ions such as Sb^3+^, is increased in the null mutants. Although ethanol is known to cause protein misfolding, the exact effects of Sb^3+^ on *Leishmania* cells is not known. The results hint at a possible effect of antimony on proper protein folding, a view that is enforced by findings that HSP90 expression rates correlate with Sb^3+^ resistance (Alexandratos et al., 2013; Vergnes et al., 2007). It remains to be seen how replacement of other ACD-protein-coding genes in *L. donovani*, i.e. *HSP20* and *P23*, will affect viability, stress tolerance and infectivity to macrophages.

In keeping with its role in protecting *Leishmania* against temperature stress, *HSP23* expression is significantly upregulated in axenically grown amastigotes, both at the RNA and protein level. This is mirrored by the absolute requirement for HSP23 in the mammalian stage. Only a few other HSPs in *L. donovani* show a similar stage-dependent upregulation – HSP100 (Hübel et al., 1995; Hübel et al., 1997; Krobitsch et al., 1998), CPN60.2 (Schlüter et al., 2000) and CPN10 (Zamora-Veyl et al., 2005). At least for HSP100, the expression profile correlates with a stage-specific role (Hübel et al., 1997; Krobitsch and Clos, 1999; Silverman et al., 2010). Other major chaperones only show a transient induction of synthesis (Brandau et al., 1995; Rosenzweig et al., 2008) during promastigote to amastigote differentiation, resulting in minor changes of their concentration in keeping with their ubiquitous role.

The changed requirement for HSP23 at elevated temperatures is also demonstrated by its altered subcellular localisation, as observed by indirect immunofluorescence. Under heat stress and in axenic amastigotes, HSP23 relocates to the perinuclear region, showing increased colocalisation with the chaperone HSP70, another suspected mediator of stress tolerance. A relocation to a nuclear or perinuclear locale is not uncommon for sHSPs. Human HSP28 (also known as HSPB1) (Arrigo et al., 1988), *Drosophila melanogaster* HSP27 (Beaulieu et al., 1989), and shrimp HSP26 (Willsie and Clegg, 2002) have all been shown to associate with nuclear structures during heat stress, the latter colocalising with HSP70, much like *L. donovani* HSP23.

Future experiments should be directed to the identification of target structures for HSP23. The native gradient gel electrophoresis already indicates a variety of apparent oligomeric complexes that contain HSP23, a variety that cannot be explained alone by the defining ability of sHSPs to form homo-oligomers (Haslbeck et al., 2005) and hence might reflect target protein interactions.

Although HSP23 appears to fulfil most of the criteria for sHSPs, the protein and its suspected homologs in other Trypanosomatidae display a less than typical domain structure, grouping with P23-like members of the family, unlike the putative HSP20. This clade of atypical sHSPs is so far restricted to kinetoplastid protozoa, an ancient branch of the eukaryota, and might have emerged from the P23 line of co-chaperones to provide functions specific to these protists. Given the structural differences from mammalian small HSPs and the essential role during the intracellular stage of *Leishmania*, steps should be initiated to screen for specific inhibitors. In addition, the role of HSP23 orthologs in other kinetoplastid protozoa (e.g. *T. brucei* and *T. cruzi*) is of interest, where heat stress is not in itself a differentiation signal.

## MATERIALS AND METHODS

### *L. donovani* culture

Promastigote stages of *L. donovani* 1SR (Rosenzweig et al., 2008) were grown at 25°C and cultured as described previously (Krobitsch et al., 1998). After electroporation, the recombinant parasites were cultivated in supplemented M199+ medium with the appropriate selection antibiotics. For routine cultivation, cells were grown to late log phase in order to maintain exponential growth. Cell density was measured using a CASY cell counter (Roche, Penzberg, Germany).

The specific IC_50_ concentrations were determined for absolute ethanol (Roth, Karlsruhe, Germany), antimonyl tartrate (Sigma-Aldrich, München, Germany) and geldanamycin (CAYLA-Invivogen, Toulouse, France).

### Electrotransfection and selection

Electrotransfection of *L. donovani* promastigotes was carried out as described previously (Ommen et al., 2009). Parasites were taken from a late log phase culture, washed twice in ice-cold phosphate-buffered saline (PBS), once in electroporation buffer and suspended at a density of 1×10^8^ ml^−1^ in electroporation buffer (Kapler et al., 1990; Laban and Wirth, 1989). 2 µg of linear DNA or 50 µg of circular DNA was used for the transfection. The DNA was mixed on ice in a 4-mm electroporation cuvette with 0.4 ml of the cell suspension. The mixture was immediately subjected to electroporation using a Gene Pulser apparatus (BioRad, München, Germany). Electrotransfection was carried out by three pulses at 2.750 V/cm and 25 µF. Following electroporation, cells were kept on ice for 10 min before they were transferred to 10-ml drug-free medium. The antibiotics G418 (50 µg ml^−1^, Roth, Karlsruhe, Germany), puromycin (25 µg ml^−1^, Sigma-Aldrich, München, Germany) and ClonNat (150 µg ml^−1^, Werner Bioreagents, Jena, Germany) were added after 24 h. Mock transfection was performed in identical fashion, without DNA, to obtain negative control cultures for antibiotic selection.

For cloning, promastigotes were seeded in supplemented M199+ at 0.5 cells per well in microtitre plates. After 10–14 days, wells positive for promastigote growth were identified and the clonal cells were transferred to culture flasks.

### *In vitro* stage differentiation

Axenic amastigotes of *L. donovani* 1SR were generated as described previously (Krobitsch et al., 1998). For this purpose, the differentiation of promastigotes towards axenic amastigotes was induced by a combination of 37°C cultivation temperature and a time-coordinated acidification of the culture medium to pH 5.5. Axenic amastigotes were cultivated at 37°C under 5% CO_2_. For amastigote quantification, the CASY Cell Counter was run in cumulative cell volume mode to include cell clusters. Equal cell volume aliquots were applied for SDS-PAGE and western blot, were quantified with RT-qPCR or were used for indirect immunfluorescence microscopy.

### *In vitro* infection experiments

Murine bone marrow macrophages (BMMs) were isolated from the femurs and tibias of C57BL/6 mice and matured in Iscove's modified Dulbecco's medium (IMDM) supplemented with 10% heat-inactivated FCS, 5% horse serum and 30% L929 supernatant (Reiling et al., 2010). The extraction of bone marrow cells from sacrificed mice was performed in compliance with Paragraph 10a, German Animal Protection Act. For infection experiments, BMMs were harvested, washed and seeded into the wells of eight-well chamber slide (NUNC, Roskilde, Denmark) at a density of 2×10^5^ cells per well and incubated for 48–72 h at 37°C under 5% CO_2_. Adherent BMMs were infected in a ratio of 1∶10 (2×10^6^) with stationary-phase promastigotes. After 4 h of incubation at 37°C in supplemented M199+, free parasites were removed by multiple washings with PBS, and incubation was continued for another 44 h in IMDM at 37°C under 5% CO_2_. The medium was removed and the cells were washed twice with PBS. Finally, the infected BMMs were fixed with ice-cold methanol. Intracellular parasites were quantified by use of DAPI (1.25 µm ml^−1^, Sigma-Aldrich, München, Germany) staining of the nuclei (*L. donovani* and BMM) and kinetoplasts (*L. donovani*). The stained cells were analysed by fluorescent microscopy. The percentage of infection and number of parasites per macrophage were counted for 200 macrophages per experiment.

### Construction and preparation of recombinant DNA

Approximately 1000 bp of 5′ non-coding DNA (5′NC) and 3′ non-coding DNA (3′NC) of the HSP23 gene (LinJ.34.0230) were amplified enzymatically from genomic *L. donovani* DNA with primers that added *Eco*RI and *Kpn*I sites (5′NC) or *Bam*HI and *Hin*dIII sites (3′NC) to upstream and downstream ends. *Swa*I sites were introduced to flank the constructs. The 5′NC amplificates were digested with *Eco*RI and *Kpn*I and ligated into a pUC19 plasmid, digested with the same enzymes. The resulting plasmids were digested with *Bam*HI and *Hin*dIII, and the 3′NC amplificates, cut with the same enzymes, were ligated between those sites. Subsequently, pUC19-HSP23-5′-3′NC was cut with *Kpn*I and *Bam*HI. Neomycin resistance and puromycin resistance genes were amplified with primers that added *Kpn*I and *Bam*HI sites to their 5′ and 3′ ends. Cut with those enzymes, the resistance markers were ligated into pUC-HSP23-5′-3′NC to yield pUC-HSP23-5′neo3′ and pUC-HSP23-5′puro3′ (supplementary material Fig. S2A). After construction, the plasmids were amplified in *E. coli*, purified by CsCl density gradient ultracentrifugation as described previously (Sambrook and Russell, 2001), and linearised with *Swa*I, to yield the constructs depicted in supplementary material Fig. S2B. The fragments containing the recombination cassette were separated by agarose gel electrophoresis and purified using the Machery and Nagel NucleoSpin Extract II Kit.

The pCLN plasmid was as described previously (Hombach et al., 2013). To create pCLS, the neomycin resistance gene was replaced with a nourseothricin resistance marker, amplified from pIRmcs(+) (Hoyer et al., 2004) to create pCLS. HSP23 (LinJ.34.0230) or P23 (LinJ.35.4540) coding sequences were amplified introducing *Kpn*I and *Bam*HI sites, respectively, to the 5′ and 3′ ends, and then fusing these into pCLS between the matching restriction sites to create pCLS-HSP23 and pCLS-P23 (supplementary material Fig. S2C).

### Genomic DNA preparation and PCR

Mutant genotypes were verified by PCR analysis of genomic DNA. For the preparation of genomic DNA the Gentra Systems Puregene Tissue Core Kit A (Qiagen, Hilden, Germany) was used, following the manufacturer's instructions.

The quality of isolated gDNA, the gene replacement and the overexpression of HSP23 were verified by PCR using the primers listed below. PCR products were separated by agarose gel electrophoresis and visualised using ethidium bromide staining. Primers used were as follows: HSP23-specific-fwd, 5′-ATGTCCACCAGCGGCCCA-3′ and HSP23-specific-rev, 5′-CTCGAGGAGGACACGTGA-3′; and P23-specific-fwd, 5′-ATGTCTCACCTTCCGATC-3′, and P23-specific-rev, 5′-TTACGCGTTGAGATCGCTG-3′.

### Expression profiling

Semi-quantitative real-time RT-qPCR was performed essentially as described previously (Choudhury et al., 2008). HSP23-gene-specific primers were HSP23-F42 (5′-CCATTCCTGGATTTGGCTCGG-3′) and HSP23-B36 (5′-GCATATCGCCGTCGTCATCTCC-3′). HSP23 mRNA abundance was calculated relative to the *Leishmania* actin signal.

### Recombinant protein expression in *E. coli* and protein purification

For the expression of antigen, the coding regions for HSP23 and P23 were amplified using specific primers that added *Nde*I sites and *EcoR*I sites to the 5′ and 3′ ends, respectively. The amplification products were cleaved with *Nde*I and *Eco*RI and ligated into the expression vector pJC45 (Schlüter et al., 2000). The expression plasmids were transformed into the bacterial strain BL21(DE3) (pAPlacIQ) and the His-tagged proteins isolated from bacterial lysates were purified using HisBind Resin (Novagen, Madison, WI) as described previously (Clos and Brandau, 1994).

The purified proteins were then used to immunise laying hens. The IgY were purified from the egg yolk as described previously (Hübel et al., 1995; Polson et al., 1985; Polson et al., 1980). The purified antibodies were tested against recombinant HSP23 or P23 and the lysates of different *Leishmania* species. The laying hens were treated and kept in accordance with Paragraph 10a of the German Animal Protection Law.

Mouse antisera against HSP70, HSP90 and HSP100 were described previously (Ommen et al., 2010).

### Western blot analysis

Production of SDS cell lysates, discontinuous SDS-PAGE and western blotting were performed according to standard protocols. Membranes were treated with blocking solution (5% milk powder and 0.1% Tween 20 in Tris-buffered saline), before they were probed with anti-HSP23 (1∶500 in blocking solution), anti-P23 (1∶200 in blocking solution) and mouse anti-α-tubulin (1∶4000 in blocking solution, Sigma-Aldrich, München, Deutschland), followed by incubation with anti-chicken-IgY antibody conjugated to alkaline phosphatase (1∶2000 in blocking solution, Dianova, Hamburg, Germany) or anti-mouse-IgG-alkaline phosphatase (1∶2000 in blocking solution, Dianova, Hamburg, Germany) as secondary antibody. Blots were developed using Nitro Blue tetrazolium chloride (Roth, Karlsruhe, Germany) and 5-bromo-4-chloro-3-indolyl phosphate (Roth, Karlsruhe, Germany). Protein bands were quantified using the software ImageJ 1.42q.

### Native PAGE

Extraction of non-denatured *Leishmania* proteins and native gradient gel electrophoresis was performed as described previously (Westwood et al., 1991). In brief, 2×10^7^ log phase promastigotes and 24-h heat-shocked promastigotes were harvested by centrifugation, washed twice with PBS, and resuspended in 40 µl extraction buffer (15% glycerol, 0.5 mM 1,10-phenanthroline, 10 mM Tris-HCl pH 8.0, 70 mM KCl). After two freeze and thaw cycles, cell lysates were cleared by centrifugation. The supernatants, containing the soluble protein fractions, were mixed 1∶5 with loading buffer (50% glycerol, 0.1% Bromphenol Blue).

The samples were run alongside a high-molecular-mass protein marker for native gels (GE Healthcare Bio-Sciences, Pittsburgh, PA) on a 6–20% polyacrylamide gradient gel in 0.5× TBE buffer. Electrophoresis was allowed to proceed for 24 h at 4°C, at 20 V/cm. This long duration of electrophoresis was necessary for all proteins to migrate to their exclusion limit regardless of their net charge (Andersson et al., 1972; Clos et al., 1990). After that, the high-molecular-mass protein marker was separated from the gel and stained with Coomassie Blue R-250. The gel was incubated at 60°C for 1 h in transfer buffer (48 mM Tris, 39 mM glycine, 0.5% SDS, 10 mM DTT), followed by western blot analysis with a specific anti-HSP23 antibody and an anti-HslV antibody (Chrobak et al., 2012; 1∶1000 in blocking solution).

### Immunofluorescence and confocal microscopy

Log phase promastigotes, heat-shocked promastigotes and axenic amastigotes (1×10^7^ cells) were sedimented, washed twice with PBS and suspended in 1 ml of PBS. Aliquots of the suspension (2×10^5^ cells) were applied on microscopic slides. After fixing the cells for 2 min in ice-cold methanol, the slides were air-dried for 20 min. Non-adherent cells were removed by a gentle wash (0.1% Triton X-100 in PBS) followed by incubation in blocking solution (2% BSA, 0.1% Triton-X 100 in PBS). Slides were then incubated for 1 h with chicken anti-HSP23 antibody (1∶100 in blocking solution) and with either mouse anti-HSP70 (1∶125 in blocking solution), mouse anti-HSP90 (1∶250 in blocking solution) or mouse anti-HSP100 (1∶100 in blocking solution) antibodies. Cell were washed three times and than incubated for 1 h with anti-chicken-IgG antibody conjugated to Alexa Fluor 594 (Dianova, Hamburg, Germany, 1∶ 500), with anti-mouse-IgG antibody conjugated to FITC (Dianova, Hamburg, Germany, 1∶250) and DAPI (Sigma-Aldrich, München, Germany, 1∶25). After washing the slides three times, Mowiol and coverslips were applied and the slides were left to dry for 24 h at 4°C. Fluorescence microscopy was carried out on an Olympus FluView1000 confocal microscope (SIM-scanner and spectral detection).

### Scanning electron microscopy

*L. donovani* cells were washed twice in PBS, fixed in 2% glutaraldehyde in sodium cacaodylate buffer and post-fixed with 1% osmium tetroxide. Samples were dehydrated at increasing ethanol concentrations (30–100%). After critical point drying, samples were treated with gold and analysed on a Philips SEM 500 electron microscope. Images were taken using a conventional 35-mm camera, and the developed black-and-white films were digitalised using a HAMA 35-mm film scanner (Monheim, Germany).

### *In silico* procedures

DNA and protein sequence analysis was performed using the MacVector® software version 12.x. Amino acid sequence alignments and tree building were performed using the MUSCLE algorithm (Edgar, 2004a; Edgar, 2004b) as part of the MacVector package.

Numerical data were analysed using the Prism® software (version 5). Greyscale and colour images were optimised for contrast using Photoshop® CS3 (Adobe) or ImageJ (NIH). Composite figures were assembled using the Intaglio® software (Purgatory). Significance was determined using the Mann–Whitney U-test.

## Supplementary Material

Supplementary Material
