# Supplementary material for: A small heat shock protein is essential for thermotolerance and intracellular survival of Leishmania donovani
Source: J Cell Sci. 2014 Nov 1;127(21):4762–73. doi: 10.1242/jcs.157297 (PMC4215717; doi:10.1242/jcs.157297)
Supplement: Supplementary Material [file supp_127_21_4762__index.html]

A small heat shock protein is essential for thermotolerance and intracellular survival of Leishmania donovani — Supplementary Material 

# A small heat shock protein is essential for thermotolerance and intracellular survival of *Leishmania donovani*

## JCS157297 Supplementary Material

**Files in this Data Supplement:**

- **Supplementary Material**
