# Supplementary material for: A small heat shock protein is essential for thermotolerance and intracellular survival of Leishmania donovani
Source: J Cell Sci. 2014 Nov 1;127(21):4762–73. doi: 10.1242/jcs.157297 (PMC4215717; doi:10.1242/jcs.157297)
Supplement: Supplementary Material [file supp_127.21.4762_JCS157297.pdf]

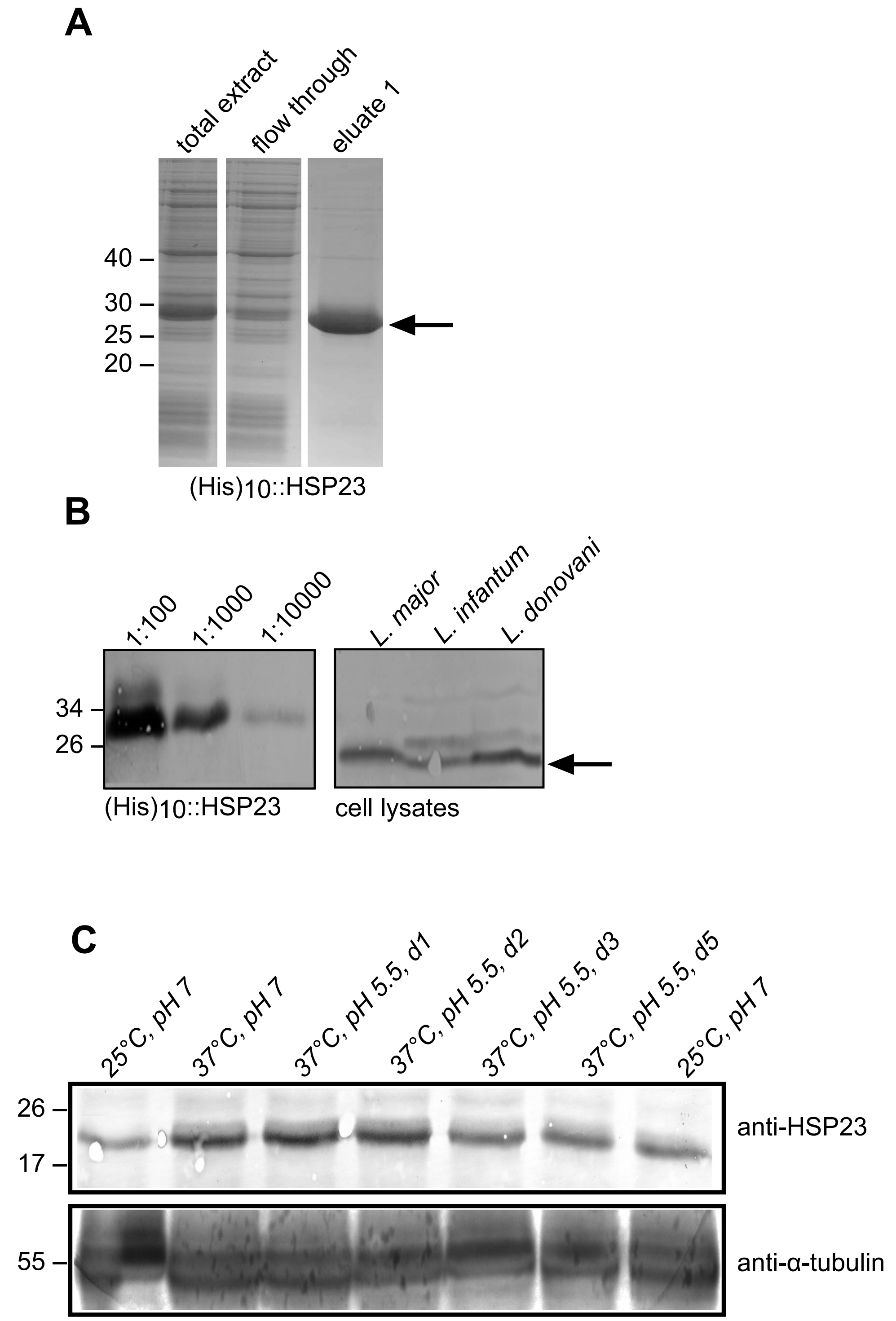

Figure S1. Purification of recombinant HSP23 and antibody production. His_{10}::HSP23 was purified from *E. coli*. Aliquots from total extract, column flow through and imidazole eluate were separated by SDS-PAGE and stained with Coomassie Brilliant Blue R (A). His_{10}::HSP23 was used to raise antibodies in chicken. Western blot analysis showed that the purified antibodies recognise as little as 0.1 ng of the recombinant protein (B, lanes 1-3) and the natural protein in the lysates of *L. major*, *L. infantum* and *L. donovani* (B, lanes 4-6). Lysates collected during *L. donovani* stage conversion were incubated with the specific anti-HSP23 antibody (C, upper panel) and as loading control with an anti-*alpha*-tubulin antibody (C, lower panel).

Supplementary Data

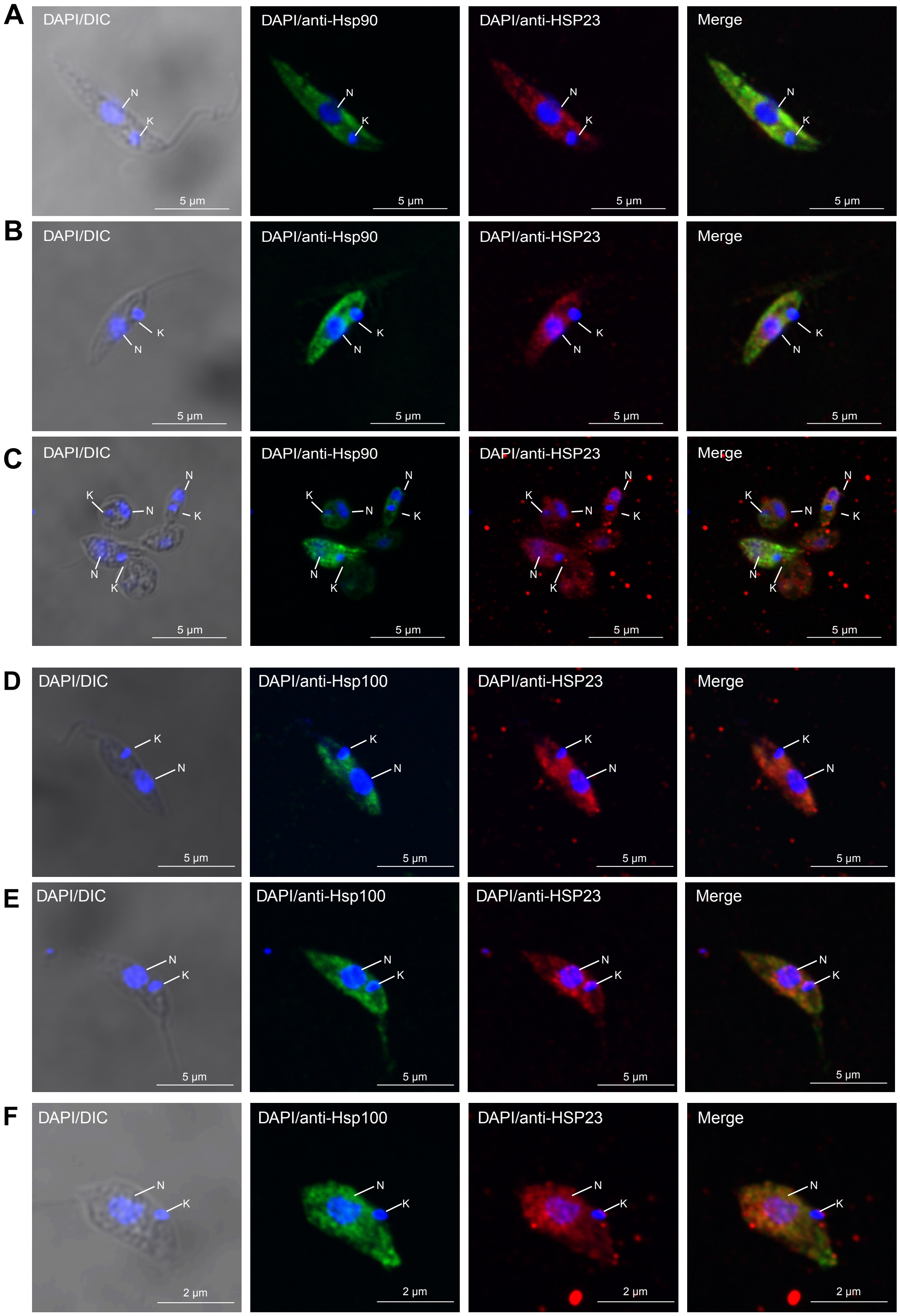

Figure S2. Comparative subcellular localisation analysis. Log phase promastigotes at 25°C (A,D) or 37°C (B,E), and axenic amastigotes (C,F) were stained with mouse anti-HSP90 (A-C, 1:250) or mouse anti-HSP100 (D-F, 1:100), chicken anti-HSP23 (1:100) and DAPI (1:25). Images were taken by confocal laser microscopy and in differential interference contrast (DIC). Representative cells from each culture were visualised as overlays of DAPI/DIC, DAPI/anti-HSP90 or DAPI/anti-HSP100, DAPI/anti-HSP23 and DAPI/HSP90/HSP23 or DAPI/HSP9100/HSP23 ("Merge"). The size bar represents size standards in (μm). k, kinetoplast; n, nucleus

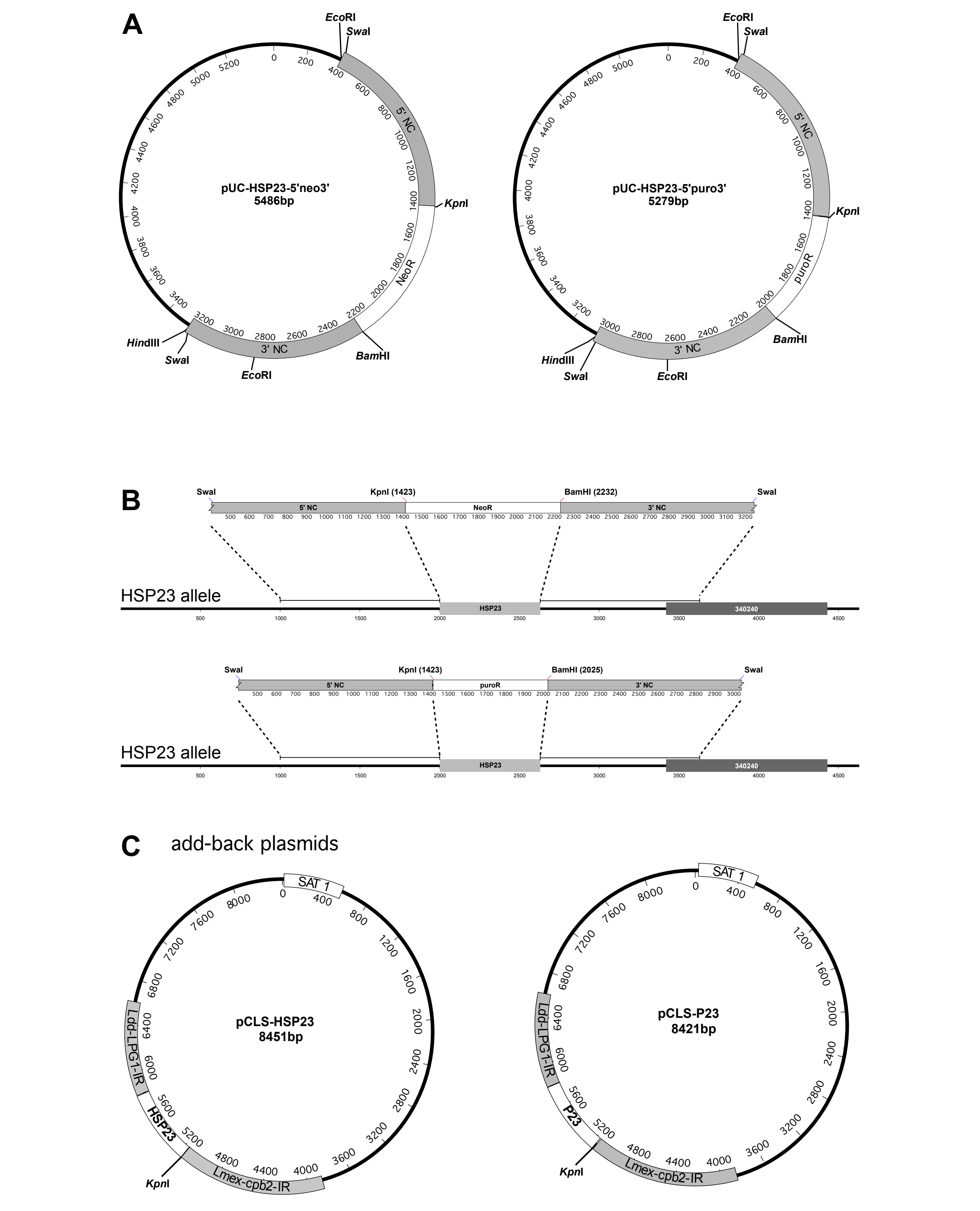

Figure S3. Schematic representation of gene constructs. (A) Plasmids constructed for HSP23 gene replacement. (B) Schematic depiction of homologous recombination between the *Swa*I-linearised replacement constructs and the HSP23 gene alleles. (C) Episomal transgenes for expression of HSP23 and P23 under nourseothricine (SAT) selection.

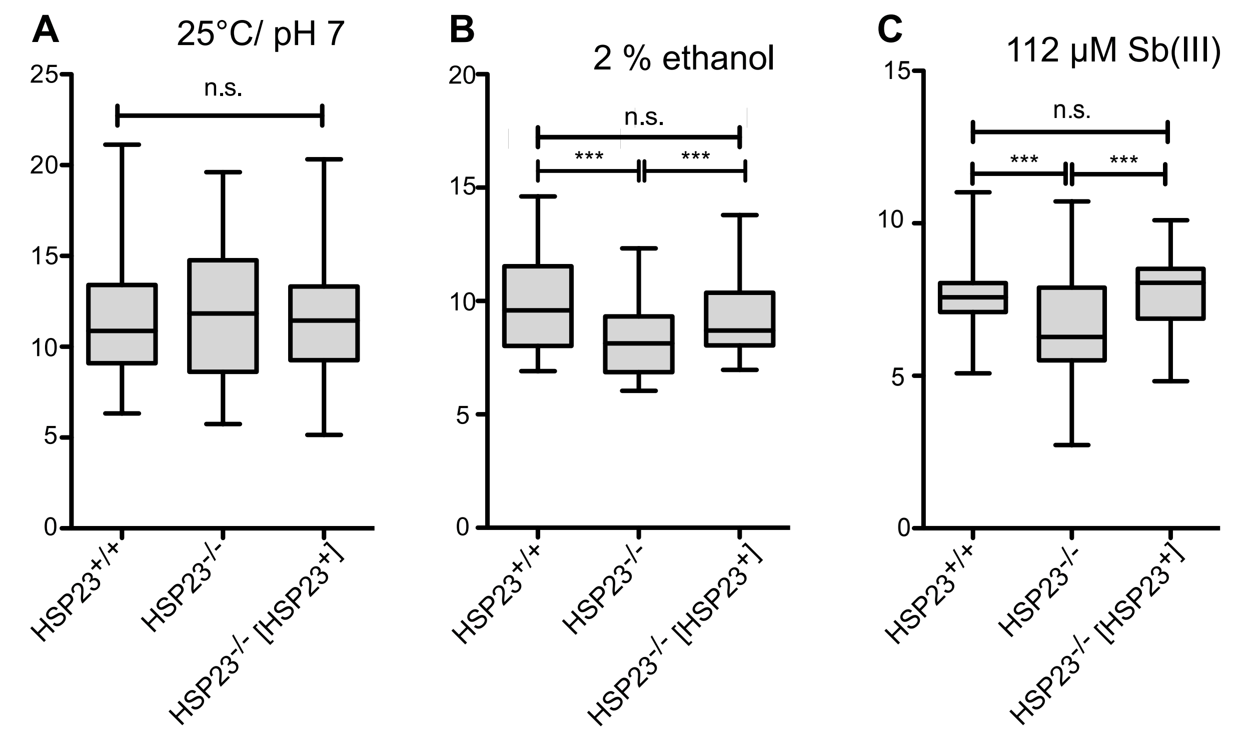

Figure S4. Cell body length analysis. *L. donovani* wild type (HSP23$^{+/+}$), HSP23 null mutant (HSP23$^{-/-}$) and null mutant with pCLS-HSP23 transgene (HSP23$^{-/-}$ [Hsp23^{+}]) were cultivated for 72 h under standard culture condition in Medium199 (A) or in Medium199 supplemented with 2% ethanol (B) or 112 μM Sb(III) (C). Cells were then fixed and visualised by scanning electron microscopy (SEM). SEM images were then imported into the Intaglio vector graphics software for relative cell body length measurements (Hombach et al., 2013). The boxes display the mean and the 25th and 75th percentiles, and the whiskers show the maxima and minima. *** = $p < 0.001$; ns = non-significant.
